# Estimation of Genetic Parameters of Growth Traits in the Inner Mongolia White Cashmere Goat (Erlangshan Type)

**DOI:** 10.3390/ani15111652

**Published:** 2025-06-03

**Authors:** Yue Shi, Yan Liu, Yunpeng Qi, Youjun Rong, Xiaofang Ao, Mingzhu Zhang, Qincheng Xia, Yanjun Zhang, Ruijun Wang

**Affiliations:** 1 College of Animal Science, Inner Mongolia Agricultural University, Hohhot 010018, China; 2 College of Vocational and Technical, Inner Mongolia Agricultural University, Baotou 014109, China; 3 Erlangshan Ranch of Inner Mongolia Beiping Textile Co., Ltd., Bayannur 015000, China; 4 Key Laboratory of Mutton Sheep Genetics and Breeding, Ministry of Agriculture, Hohhot 010018, China; 5 Key Laboratory of Goat and Sheep Genetics, Breeding and Reproduction in Inner Mongolia Autonomous Region, Hohhot 010018, China; 6 Inner Mongolia Key Laboratory of Sheep & Goat Genetics, Breeding and Reproduction, Hohhot 010010, China

**Keywords:** growth traits, variance components, genetic parameters, Inner Mongolia white cashmere goats

## Abstract

This study aimed to estimate the variance, heritability, and genetic correlation of growth traits in Inner Mongolia white cashmere goats (Erlangshan type) (IMWCG-ER). Data collected from the Erlang Mountain Ranch in 2022–2023 were analyzed. The traits studied included birth weight (BW), weaning weight (WW), pre-weaning daily gain (ADG), and the 12-month weight (12W). In animal models, single and multi-trait analyses were performed using the restricted maximum likelihood (REML) method to estimate genetic parameters, and six different models were fitted for each trait by ignoring or including maternal permanent environmental effects, maternal additive genetic effects, and the interaction with individual additive genetic effects. The accuracy and suitability of each model were tested using the likelihood ratio and AIC and BIC tests. The heritability estimates of birth weight, weaning weight, daily gain before weaning, and the 12-month weight were 0.3884, 0.2951, 0.2749, and 0.2192, respectively. The absolute value of the genetic correlation coefficient between traits ranged from −0.8700 to 0.5529. The highest genetic association was between birth weight and pre-weaning daily gain (−0.8700), followed by birth weight and the 12-month weight (−0.6256). The absolute value of phenotypic correlation coefficients ranged from −0.7906 to 0.3562. The highest phenotypic correlation was between birth weight and daily gain before weaning, and the correlation coefficient was −0.7906.

## Simple Summary

The Inner Mongolia white cashmere goat (Erlangshan type) is a local dual-purpose goat breed of cashmere and meat in China. The meat it produces has high nutritional value. Genetic parameter estimation is a basic work in livestock breeding. The accurate estimation of population genetic parameters plays a very important role in studying and revealing the genetic mechanisms of quantitative traits, discussing the breeding effect, accurately assessing the breeding value of breeding animals, formulating a selection scheme, calculating genetic progress, and formulating a breeding plan. The aim of this study was to investigate the genetic factors affecting the growth traits of Inner Mongolia white cashmere goats (Erlangshan type) and to accurately evaluate the genetic parameters to provide the key basis for the genetic improvement of this breed. The results showed that it would be possible to obtain improvements in the pre-weaning traits of Inner Mongolia white cashmere goats (Erlangshan) if incorporated into the selection program.

## 1. Introduction

China plays a pivotal role in cashmere goat breeding, housing renowned breeds including the Liaoning, Inner Mongolia white, Shanbei white, and Yanshan cashmere goats [1]. As China’s economy grows rapidly and people’s living standards continue to improve, the demand for livestock products has also surged and become more diversified. The cashmere goat industry places great emphasis on both the quality and yield of cashmere. As a dual-purpose breed for both cashmere production and meat utilization, the Inner Mongolia white cashmere goat has garnered the attention of breeders. They are well aware of the direct correlation between the body weight of these goats and the breeding profits, which has become a crucial factor in their breeding and management strategies.

Cashmere goat products face intense competition in the global market. Simply having high-quality cashmere or a high meat yield is no longer sufficient. There is a growing trend towards finer cashmere and higher meat production. This makes growth traits a key indicator of cashmere goats’ production performance [2]. The Inner Mongolia white cashmere goat, prized for its meat and cashmere, holds a unique position in global animal husbandry due to its strong resistance to drought, cold, diseases, and adaptability to rough feed. It provides both farmers with high-quality cashmere fiber and herders with ample meat resources [3]. The average weight of bucks is 80 kg, while does weigh approximately 50 kg. The meat from goats is known for being delicious, tender, and juicy, with no unpleasant odor. It is easy to digest, rich in essential amino acids, and low in fat and cholesterol. Local herdsmen use this meat both as food and for medicinal purposes [4].

Heritability, repeatability, and correlation are key parameters in animal breeding research and the design and implementation of breeding programs [5,6]. These parameters can be used to detect variation in the population, allowing for the determination of subsequent breeding measures. Estimating heritability and predicting breeding values are essential for selecting optimal mating strategies and enhancing selection responses [7]. Growth and development traits are the most important traits in livestock breeding strategies and are influenced by various factors, which can be categorized into genetic and non-genetic factors, and their interactions [8,9]. Non-genetic factors, such as the year of birth, the age of does at suckling, and the level of feeding and management, are fundamental in determining the growth and development of livestock and should not be overlooked. Genetic factors encompass the impact of an animal’s individual genetic effects, maternal genetic effects, maternal permanent environmental effects, and the effects of the interactions between these components [10]. This helps determine the extent to which these traits can be enhanced through artificial selection. Genetic evaluation also makes it possible to accurately determine the genetic and phenotypic correlations between different traits. This information can elucidate the intrinsic relationships between traits affected by genetic factors, predict responses to indirect selection, and analyze the genetic structure of complex traits [11]. Furthermore, it provides a theoretical foundation for selecting multiple traits and optimizing breeding programs for cashmere goats. The accurate estimation of genetic parameters is essential for improving populations through genetic methods. These estimates are crucial for developing effective genetic improvement plans [12]. The extent to which different traits are influenced by genetic factors and the relationships between traits can be clarified by accurately assessing parameters such as heritability and genetic correlation. This, in turn, allows for the determination of specific selection strategies for different traits. However, the accuracy of the estimates varies according to the production system, the traits under selection, and the statistical models employed [13,14]. Traits with moderate to high heritability indicate that these traits are significantly influenced by genetic factors, and significant genetic progress can be achieved within a relatively short period through selection [15]. In contrast, low heritability traits are greatly affected by environmental factors, and it is difficult to achieve ideal genetic progress by relying solely on traditional selection methods.

It is necessary to adopt different comprehensive breeding strategies for different traits, such as optimizing feeding and management conditions, enhancing environmental factors, and so on, to reduce the influence of the environment on trait performance. Using indirect selection methods in combination with other strategies, low heritability traits can be indirectly improved by selecting high heritability traits that are highly correlated with low heritability traits [16]. Information on genetic correlation is helpful in determining the tradeoff between traits in the breeding process and realizing the joint improvement of multiple traits [17]. For traits with a positive genetic correlation, selecting one of the traits will result in the co-selection of the other trait. For traits with a negative genetic correlation, selection for one of the traits will result in an adverse selection for the other trait.

Breeders need to determine effective breeding strategies based on their needs and objectives [18]. Genetic parameter estimation is an important basis for animal breeding and genetic improvement [19]. It provides important information for livestock breeding, and has a significant impact on promoting the sustainable development of the animal industry. Given the lack of reports on the genetic parameter estimation of growth and development traits in the Inner Mongolia white cashmere goat (Erlangshan type), this study aimed to evaluate these parameters. The overarching goal was to enhance the growth rate, meat production performance, and international competitiveness of the Inner Mongolia white cashmere goat (Erlangshan type) and to provide a solid theoretical foundation for its breeding and genetic improvement. The overarching goal was to enhance the growth rate, meat production performance, and international competitiveness of the Inner Mongolia white cashmere goat (Erlangshan type) and to provide a solid theoretical foundation for its breeding and genetic improvement. Overall, this study fills a research gap in genetic parameter estimation for this breed and has far-reaching implications for the entire cashmere goat breeding sector.

## 2. Materials and Methods

### 2.1. Data Collection and Studied Traits

This study’s data were obtained from 8409 kids’ records at Erlangshan Ranch, Inner Mongolia Beiping Textile Co., Ltd. (Bayannur, China), from 2020 to 2023. Excel 2019 was used to organize data, check the integrity of pedigree data, and exclude individuals with incomplete information. Then, the dataset was screened, excluding records with over three times the standard deviation. After data processing, the birth weight (BW) records of 4818 newborn goats born to 47 rams and 2353 does, weaning weight (WW) and pre-weaning daily gain (ADG) records of 4236 kids born to 47 rams and 2217 does, and the 12-month weight (12W) records of 775 goats born to 30 rams and 667 does were utilized. Trait data were obtained via on-site measurements. BW was measured right after birth using a 0.01 accurate scale, noting birth time and ear tag number. WW was measured on an empty stomach at a uniform weaning age, adjusted to a 90-day standard. ADG was calculated as (weaning weight − birth weight)/pre-weaning days. The 12W was measured on an empty stomach at 12 months, adjusted to a 360-day standard. All the body weights at all stages were measured using an ND3011-S livestock scale. As shown in Table 1, the average BW, WW, ADG, and 12W for rams were approximately 2.05, 1.91, 1.61, and 1.61, respectively. For does, trait values varied from 25.83 to 102.51. A thorough pedigree check verified sufficient data for estimating genetic components. Table 1 shows that the CVs of BW, WW, ADG, and 12W were 20.27%, 26.19%, 5.59%, and 13.48%, respectively. These values highlight significant trait variability, which is essential for the genetic assessment and improvement of Inner Mongolia white cashmere goats (Erlangshan type).

### 2.2. Feeding Practices

Urad Zhongqi, Bayannur City, where the Erlang Mountain Ranch is located, is located in the western part of the Inner Mongolia Autonomous Region and northeast of Bayannur, between 41°07′–41°28′ north latitude and 107°16′–109°42′ east longitude. It is located west of the Inner Mongolia Plateau, a semi-desert area dominated by desert steppe. The natural grassland vegetation is sparse and low, consisting mainly of small perennial shrubs and perennial tufted grass, and it is the national breeding farm for the Inner Mongolia white cashmere goat (Erlangshan type).

Kids are weighed at birth and marked with ear tags. From birth to 30 days of age, they are raised in pens. During this period, the kids grow and develop rapidly, but the nutritional value of breast milk gradually declines, making it difficult to maintain the nutrient supply. Therefore, in addition to breastfeeding, hay is provided during the day and the kids are allowed to eat freely, while, at night, they are kept in the same pen as the does to facilitate breastfeeding.

From 30 days until weaning, kids are nursed once in the morning and once in the evening, with each nursing period lasting two hours. Kids graze separately from their mothers during the day and are penned together with them at night. This goat ranch adopts unified weaning, supplementing with alfalfa green hay for transitional feeding. Kids are weaned and weighed at approximately 90 days of age. After weaning, the kids are transferred to breeding herds, managed in sex groups, fed through grazing and complementary feeding, and weighed at 6 and 12 months.

### 2.3. Statistical Analyses

The animal model includes fixed effects and random effects. Previous studies by our research group [20] concluded that the fixed effects affecting birth weight, weaning weight, pre-weaning daily gain, and the 12-month weight include sex, birth type, the age of does, birth month, birth group, and the interactions of these effects. In this study, ASReml-R 4 was used to estimate the variance components of each trait by fitting six single-trait animal models containing individual additive effects, maternal additive genetic effects, maternal permanent environmental effects, and individual additive and maternal additive genetic interaction effects. Finally, AIC, BIC, and LRT tests were used to determine the best model for each trait, and then the heritability was estimated. The fitting model was as follows:

(1) $$y=Xb+Z_{1}a+e$$

(2) $$y=Xb+Z_{1}a+Z_{3}c+e$$

(3) $$y=Xb+Z_{1}a+Z_{2}m+e\;\;\;\;\;\;\;\;\;\;\;\;\;COV(a,m)=0$$

(4) $$y=Xb+Z_{1}a+Z_{2}m+e\;\;\;\;\;\;\;\;\;\;\;\;\;COV(a,m)=A\sigma_{am}$$

(5) $$y=Xb+Z_{1}a+Z_{2}m+Z_{3}c+e\;\;\;\;\;\;COV(a,m)=0$$

(6) $$y=Xb+Z_{1}a+Z_{2}m+Z_{3}c+e\;\;\;\;\;\;COV(a,m)=A\sigma_{am}$$

where *y* represents the observation vector on the different traits; *b*, *a*, *m*, *c*, and *e* are the vectors of fixed effects, individual direct additive genetic effects, maternal additive genetic effects, maternal permanent environmental effects, and residual effects, respectively; *X*, $Z_{1},{\;Z}_{2}$, and $Z_{3}$ are incidence design matrices relating the fixed effects, direct additive genetic effects, maternal additive genetic effects, and maternal permanent environmental effects, respectively, to a vector of *y*. It is assumed that individual additive genetic effects, maternal additive genetic effects, maternal permanent environmental effects, and residual effects are normally distributed, with a mean value of 0. $Var\left(a\right)=A\sigma_{a}^{2},Var\left(m\right)=A\sigma_{m}^{2}$, $Var\left(c\right)=I_{d}\sigma_{c}^{2}$, $Var\left(e\right)=I_{d}\sigma_{e}^{2}$, where $\sigma_{a}^{2}$, $\sigma_{m}^{2}$, $\sigma_{c}^{2}$, and $\sigma_{e}^{2}$ are the direct additive genetic variance, maternal additive genetic variance, maternal permanent environmental variance, and residual variance, respectively. A represents the additive genetic correlation matrix, $I_{d}$ and $I_{n}$ are the identity matrices of equal order to each structural matrix, respectively, and $\sigma_{am}$ represents the covariance between the direct additive genetic effect and the maternal additive genetic effect. Finally, after obtaining the best genetic evaluation model for each trait, the genetic correlation of each trait was estimated using the animal model with no repetitive force for the two traits.

In this study, the likelihood ratio (LR) test was used to compare the goodness-of-fit of two nested models, where one model is a special case of the other, and the restricted model imposes more constraints than the unrestricted model. The likelihood ratio test is based on maximum likelihood estimation and determines which model is better by comparing the likelihood function values of the two models. The likelihood function describes the probability of observing the data given the model parameters. The likelihood ratio test statistic (*G*) is defined as follows:

(7) $$G=-a\ln(\frac{L_{R}}{L_{U}})$$

where $L_{R}$ is the maximum likelihood value of the restricted model and $L_{U}$ is the maximum likelihood value of the unrestricted model. Under the null hypothesis (the restricted model is true), when the sample size is large enough, *G* approximately follows a chi-squared distribution, with the degrees of freedom equal to the difference in the number of parameters between the two models.

Under the condition that the sample size is large enough and the two models are nested, the likelihood ratio test statistic asymptotically follows a chi-square distribution. Based on the properties of this distribution, the significance of the differences between the two models caused by different constraint conditions can be determined by comparing with the critical values of the chi-square distribution at the corresponding degrees of freedom.

The Akaike information criterion (AIC) is a criterion used to balance the goodness-of-fit and complexity of a model. It not only takes into account how well the model fits the data but also the number of parameters in the model.

The formula for AIC is

(8) AIC=2k−2ln(L)

where *L* is the maximum likelihood value of the model and *k* is the number of parameters in the model.

A smaller AIC value indicates a better model because it strikes a better balance between goodness-of-fit (measured by −2ln⁡L, where a smaller value implies a better fit) and complexity (measured by 2*k*, where more parameters mean higher complexity).

The Bayesian information criterion (BIC) is similar to the AIC and is also a criterion for model selection, considering both the goodness-of-fit and the complexity of the model. Unlike AIC, BIC penalizes model complexity more severely, especially when the sample size is large.

The formula for BIC is

(9) BIC=kln(n)−2ln(L)

where L is the maximum likelihood value of the model, k is the number of parameters in the model, and n is the sample size. Compared with AIC, the penalty term *k*$\ln\left(n\right)$ in BIC increases more rapidly as the sample size n increases, which means that BIC tends to favor simpler models when the sample size is large.

## 3. Analysis of Results

### 3.1. Variance Component Estimation

As shown in Table 2, the maximum estimated birth weight heritability was 0.3884 (model1) and the minimum was 0.0279 (model3), respectively. The highest and lowest maternal heritability estimates were 0.1220 (model6) and 0.0976 (model3), respectively. The proportion of maternal permanent environmental effects to phenotypic variance was 8.28 × 10^−6^~0.0933. The direct genetic correlation with maternal heritability was −0.0641 for model4 and −0.0660 for model6. They had a low negative correlation.

The highest and lowest heritability estimates of weaning weight were 0.2114 (model1) and 0.0270 (model3), respectively. The highest and lowest maternal heritability estimates were 0.1661 (model6) and 0.1076 (model5), respectively. The proportion of maternal permanent environmental effects to phenotypic variance was 1.7609 × 10^−8^~0.1160. The direct genetic correlation with maternal heritability was −0.0585 for model4 and −0.0586 for model6. These show that they had a low negative correlation.

The highest and lowest heritability estimates of pre-weaning daily gain were 0.7251 (model1) and 0.0057 (model3), respectively. The maximum and minimum maternal heritability were 0.1640 (model3) and 0.0954 (model5), respectively. The proportion of maternal permanent environmental effects to phenotypic variance was 0.0756–0.1784. The individual additive genetic effect and the maternal additive genetic effect had a low negative correlation, with correlation coefficients of −0.0471 (model4) and −0.0280 (model6).

The highest and lowest heritability estimates of the 12-month weight were 0.2192 (model1) and 0.1996 (model4), respectively. The highest and lowest maternal heritability estimates of model3 and model4 were 0.0276 and 0.0160, respectively. The proportion of maternal permanent environmental effects to phenotypic variance was 6.4320 × 10^−6^~0.0276. There was a low positive correlation between the individual additive genetic effect and the maternal additive genetic effect, with correlation coefficients of 0.0100 and 0.0116 for model4 and model6.

### 3.2. Eigenvalues of the Genetic Assessment Model

As shown in Table 3, the AIC and BIC values of model 1 were the largest in terms of birth weight, weaning weight, and pre-weaning daily gain. The AIC values were −3072.361, 34,479.91, and 12,920.58, while the BIC values were −3059.43, 34,492.6, and 12,933.26, respectively. The minimum AIC and BIC values of birth weight were −3246.205 (model6) and −3213.876 (model6), respectively. The minimum AIC and BIC values of weaning weight were 12,892.71 (model2) and 12,911.73 (model2). The minimum values of AIC and BIC for pre-weaning daily gain, both in model2, were 34,413.19 and 34,433.15, respectively. For the 12-month weight, the maximum value of AIC was 4314.169 (model6) and the minimum value was 4308.41 (model1); the maximum value of BIC was 4340.119 (model6) and the minimum value was 4318.79 (model1).

### 3.3. Likelihood Ratio Test

The results of the likelihood ratio test are displayed in Table 4. Significant differences were observed among the birth weight models, indicating that incorporating maternal additive genetic effects, maternal permanent environmental effects, and their interaction effects impacts the estimation of birth weight. Regarding weaning weight, there was a highly significant disparity between model1 and other models, suggesting that considering only individual additive effects is insufficient when estimating the variance of weaning weight. There was no significant distinction among the models for pre-weaning daily gain, implying that individual additive effects should be taken into account solely for determining the heritability of this trait. Concerning the 12-month-old weight, there was a substantial difference between model1 and the other four models; however, no significant difference existed between model6 and model1. This indicates that employing an overly complex model reduces accuracy when estimating heritability for the 12-month weight; thus, selecting model6 as the most suitable option is not recommended.

### 3.4. Genetic and Phenotypic Correlations

The genetic correlations between traits were estimated using the animal model, with no repetitive force for two traits; the results are summarized in Table 5. The results show that the genetic correlation between birth weight and weaning weight was 0.5529, reflecting a moderate positive correlation; with $\left|Z\right|$ > 2.58, the genetic correlation was significantly different from 0. The phenotypic correlation between birth weight and weaning weight was 0.3906, showing a weak positive correlation, and $\left|Z\right|$ > 2.58, indicating that the phenotypic correlation was also significantly different from 0. The genetic correlation between birth weight and pre-weaning daily gain was −0.8700, with $\left|Z\right|$ > 2.58, demonstrating a significant negative correlation with 0. The phenotypic correlation between birth weight and pre-weaning daily gain was −0.7906, both showing strong negative correlations, and $\left|Z\right|$ > 2.58 indicated the phenotypic correlation was significantly different from 0. The genetic correlation between birth weight and the 12-month weight was −0.6256, showing a moderate positive correlation; with $\left|Z\right|$ < 1.96, the genetic correlation was not significantly different from 0, and no true genetic association between the two traits could be confirmed. The phenotypic correlation between birth weight and the 12-month weight was 0.2014, showing a weak correlation, but $\left|Z\right|$ > 2.58 indicated the phenotypic correlation was significantly different from 0. The genetic correlation between weaning weight and pre-weaning daily gain was 0.3809, with $\left|Z\right|$ > 2.58 indicating a significant genetic correlation with 0. And the phenotypic correlation between weaning weight and pre-weaning daily gain was 0.3562, showing a moderate positive correlation, and $\left|Z\right|$ > 2.58 confirmed the phenotypic correlation was significantly different from 0. The genetic correlation between weaning weight and the 12-month weight was 0.3329; with $\left|Z\right|$ < 1.96, the genetic correlation was not significantly different from 0, and no true genetic association between the two traits could be confirmed. And the phenotypic correlation between weaning weight and the 12-month weight was 0.2497, showing a weak positive correlation, and $\left|Z\right|$ > 2.58 indicated the phenotypic correlation was significantly different from 0. The genetic and phenotypic correlation coefficients of pre-weaning daily gain and year of life were small.

## 4. Discussion

### 4.1. Model Evaluation

This study concludes that the heritability estimates of birth weight, weaning weight, pre-weaning daily gain, and 12-month weight in the optimal model are 0.1330, 0.0312, 0.0076, and 0.2192, respectively. Birth weight and 12-month weight are the traits with moderate heritability, while weaning weight and pre-weaning daily gain are the traits with low heritability. In domestic animals, growth traits are influenced not only by the genetic potential of the animal but also by maternal genetics and permanent environmental factors [21]. This is due to the excessive number of parameters and an overly complex model during the training of the model, leading to overfitting of the model and resulting in reduced stability and low reliability of the final model [19]. In particular, prior to weaning, ADG is significantly more affected by maternal permanent environmental effects than other effects, which directly leads to low individual heritability. Shorepy et al. [22] also concluded that the estimated genetic parameters will be low if maternal genetic effects are included in the model when estimating genetic parameters of early growth traits. The model comparison in this study also showed that the optimal model for birth weight, weaning weight, and pre-weaning daily gain should include maternal permanent environmental effects, maternal additive genetic effects, or their interaction effects. However, only the individual additive effect was included in the optimal model for age. Numerous studies have indicated that when estimating the heritability of post-weaning traits, the maternal additive effect and maternal permanent environmental effect are typically excluded from the model [23,24,25]. This indicates that, as kids grow up, undergo the weaning period, and gradually leave the maternal environment, the influence of maternal genetic effects and maternal permanent environmental effects gradually weaken, and the proportion of individual additive effects gradually increases.

### 4.2. Heritability

The excessive number of parameters in the model leads to its overfitting. Therefore, this study selects model1, which only includes the individual additive effect, to estimate the heritability of each trait. Finally, the heritabilities of BW, WW, ADG, and 12W are 0.3884, 0.2951, 0.2749, and 0.2192 respectively. There are similarities and differences between the results of this study and those of previous studies. The differences are caused by multiple reasons. In different studies, varying goat breeds, sample sizes, and evaluation methods may have been employed, all of which can influence the estimation of genetic parameters [26]. The genetic backgrounds of different varieties exhibit variation. Furthermore, as a result of long-term selective breeding, the allele frequencies at certain gene loci have been altered, leading to differences in the ratio of genetic variance to phenotypic variance [27]. During the transition from wild to domesticated environments, livestock have adapted to environmental variations, including climate and feeding patterns, and have been subject to human-driven selection for functional traits. Consequently, different breeds across various regions exhibit significant differences in the expression of the same traits. Moreover, the estimated values of heritability also vary to different degrees [28]. The specificity of varieties is a key factor contributing to variations in heritability estimates [29]. The sample size also limits the estimation of genetic parameters [30]. A small sample size cannot fully represent the overall variation, resulting in large deviations of the estimated value, low precision, and weak statistical power [31]. It may overestimate or underestimate heritability or misinterpret the genetic components of traits. In contrast, a large sample size can comprehensively cover the variation, making the estimated value more accurate, the precision higher, and the statistical power stronger, thus making it easier to reveal the genetic laws. Differences in environmental factors may also lead to different estimates of heritability. Different feeding and management conditions, geographical environments, and climate conditions may all impact the performance of goat traits, thus affecting the estimation of genetic parameters [32,33]. SANTOS [34] estimated the genetic parameters of Angolan Nubian goats and concluded that the heritability of birth weight under the optimal model was 0.09. The optimal model mentioned in that study was the same as model6 in the current study. Barazandeh [32] estimated that the heritability estimate of the 12-month weight of Raini goats was 0.29 and concluded that maternal permanent environment and maternal genetic effects had no effect on the 12-month weight in model comparisons. When estimating the genetic parameters of Sardi sheep, Boujenane [35] concluded that the direct heritability of birth weight was 0.07 and the maternal heritability was 0.13. In Ettawa Grade goat [36], the heritability estimates of birth weight, weaning weight, and the 12-month weight were 0.54, 0.35, and 0.68, respectively. The heritability of birth weight in Thai goats [17] was 0.41, and the correlation coefficient between individual additive effects and maternal additive effects was −0.81. Herrera [37] estimated the heritability of birth weight and the 12-month weight for five goat breeds, namely Nubia, Granadina, Saanen, Toggenburg, and Alpine. The result shows that the direct heritability estimates of birth weight were 0.25, 0.25, 0.25, 0.51, and 0.20, respectively. The covariance between individual additive effects and maternal additive effects were 0.15, 0.15, 0.24, 0.28, and 0.19, respectively. The direct heritability estimates of birth weight were 0.04, 0.13, 0.04, 0.41, and 0.15, respectively. The heritability estimates of maternal additive genetic effect and maternal permanent environmental effect were 0.03, 0.08, 0.08, 0.38, and 0.02, respectively. Before weaning, individuals are influenced by the mother’s own health level, the hormonal regulation of the mother, and the intrauterine environment. In mammals, in addition to the genes passed on to the offspring, the mother can also influence the phenotype of the offspring through the prenatal cytoplasmic and uterine environment, along with postnatal maternal behavior and milk production [38]. Studies have shown that when the mother experiences stress and secretes elevated levels of cortisol, this hormonal imbalance can suppress fetal growth [39,40,41]. Other studies have pointed out that the intrauterine environment, such as nutrient supply, oxygen content, and amniotic fluid volume, will affect the growth and development of the fetus [42,43,44,45,46,47]. Bangar [48] estimated the heritability of Harnali sheep’s birth weight to be 0.22. According to Ofori and Hagan [49], heritability estimates of the birth weight and the weaning weight of WAD goats were 0.45 and 0.57, respectively. Heritability estimates of the birth weight, weaning weight, and 12-month weight of Jamunapari goats were reported to be 0.32, 0.35, and 0.32, respectively in the study conducted by Dige [50]. Jafaroghli [51] estimated that the heritability of the weaning weight in Mogani sheep was 0.09. Zhang [52] estimated that the heritability of the birth weight of Boer goats was 0.17, the heritability of the weaning weight was 0.22, and the heritability of the pre-weaning daily gain was 0.07. Latifi and Razmkabir [53] estimated the heritability of the birth weight, weaning weight, and 12-month weight of Mrkhoz goats, and the results were 0.25, 0.09, and 0.31, respectively. According to Mohsen Gholizadeh [54], heritability estimates for the birth weight, weaning weight, and 12-month weight of Baluchi sheep were determined to be 0.05, 0.07, and 0.15, respectively. Heritability estimates of the birth weight and weaning weight of Awassi [55] sheep were 0.23 and 0.168, respectively. The results of these studies are quite different from those reported in the literature, which may be due to the sample size, model selection, environment, and population structure. The above studies showed that the heritability of birth weight was significantly higher than that of the weaning weight.

### 4.3. Genetic and Phenotypic Correlations

The results of this study showed that the phenotypic correlations between birth weight and weaning weight, birth weight and pre-weaning daily gain, and birth weight and the 12-month weight were 0.3906, −0.7906, and 0.2014, respectively, while the genetic correlations were 0.5528, −0.8700, and −0.6256, respectively. The phenotypic correlations between WW and ADG and WW and 12W were 0.3652 and 0.2497, respectively, while the genetic correlations were 0.3809 and 0.3329, respectively. The phenotypic and genetic correlations between ADG and 12W were 0.0993 and 0.0755, respectively. Snyman [56] concluded that the phenotypic and genetic correlations between the birth weight, weaning weight, and 12-month weight showed moderate positive correlations. Gopal [57] also showed that there was a moderate positive genetic correlation between the birth weight and weaning weight, pre-weaning daily gain, and 12-month weight in Sirohi goats, and a moderate positive phenotypic correlation between the birth weight and weaning weight and the 12-month weight, but a low phenotypic correlation between the birth weight and pre-weaning daily gain. Strong positive correlations were found between the weaning weight and pre-weaning daily gain and the 12-month weight. Phenotypic and genetic correlations were also identified between the pre-weaning daily gain and 12-month weight. Rashidi [58] investigated the phenotypic and genetic correlations between the birth weight, weaning weight, and pre-weaning daily gain in Markhoz goats, concluding that these traits exhibited a moderately high positive correlation. The genetic and phenotypic correlations between the birth weight and pre-weaning daily gain in Sardi sheep [35] were 0.23 and −0.01, respectively. The study by Ankit [59] showed that the genetic and phenotypic correlations between the birth weight and weaning weight were relatively not strong. In contrast, the genetic and phenotypic correlations between the birth weight and pre-weaning daily gain were −0.14 and 0.07, respectively, and the genetic and phenotypic correlations between the birth weight and weaning weight were −0.68 and −0.11, respectively, both indicating negative associations. The negative correlation between the birth weight and pre-weaning daily weight gain has been noted. These studies pointed out that a fetus with an excessively high birth weight increases the risk of difficult labor, thereby raising the possibility of maternal hemorrhage and the potential risk of neurological damage or fractures in the newborn [4]. Cubs with low birth weight are capable of more efficiently absorbing nutrients from breast milk to fulfill their developmental requirements. Additionally, the oligosaccharides present in breast milk function as prebiotics, supporting and enhancing the development of microbial communities and immune function within the cubs’ bodies [60]. On the other hand, low birth weight induces alterations in hormonal levels within the offspring, including the enhanced secretion of insulin-like growth factor (IGF). IGF plays a critical role in promoting the growth and repair of tissues such as muscle and bone, thereby contributing to an increase in the offspring’s body weight [61]. There may also be complex interactions between genes regulating birth weight and pre-weaning daily gain that need further exploration in future studies.

## 5. Conclusions

When estimating the genetic parameters of pre-weaning traits, both maternal genetic effects and maternal permanent environmental effects should be thoroughly accounted for. In contrast, when evaluating the 12-month weight, these influences can be appropriately downweighed. The variation in estimated genetic parameter values across different models highlights the critical importance of model selection for achieving accurate breeding value predictions. The heritability estimates for the traits examined in this study are relatively low, indicating that population selection based on these traits may result in slow genetic progress in growth-related traits. Finally, given the presence of genetic variation in growth traits and moderate-to-high genetic correlations among pre-weaning traits, it is feasible to incorporate improvements in pre-weaning traits of Inner Mongolia white cashmere goats (Erlangshan) into the selection program.

## Figures and Tables

**Table 1:** Characteristics of the phenotypic data of Inner Mongolia white cashmere goats.

Item	BW, kg	WW, kg	ADG, kg/d	12W, kg
Number of animals in pedigree	4889	4264	4264	1332
The number of sires	47	47	47	30
The number of dams	2353	2217	2217	667
The number of records	4818	4236	4236	775
Records with sire but no dam	15	15	15	10
Records with dam but no sire	424	379	379	127
Average number of progenies per sire	102.51	90.13	90.13	25.83
Average number of progenies per dam	2.05	1.91	1.91	1.16
Mean	2.54	15.17	0.89	23.66
SD	0.51	3.97	0.05	3.19
CV (%)	20.27	26.19	5.59	13.48

Note: BW represents the body weight of Inner Mongolia cashmere goats at birth. WW refers to the weaning weight of the goats. ADG is the average daily gain of the goats before weaning. 12W denotes the 12-month weight of the goats. The number of sires is the quantity of male parents in the pedigree. The number of dams is the quantity of female parents in the pedigree. No. of records is the total number of data records for each trait. Number of sires and number of dams are the numbers of male and female parents, respectively, involved in the data collection. Average litter size of sires and average litter size of dams are the average numbers of offspring per litter for sires and dams, respectively. Average number of progenies per sire is the average number of offspring produced by each male parent. Average number of progenies per dam is the average number of offspring produced by each female parent. Mean is the arithmetic mean value of the trait data. SD measures the variation or dispersion of a set of values. CV (%) is the coefficient of variation, calculated as the ratio of the standard deviation to the mean, expressed as a percentage.

**Table 2:** (Co)variance components of body weight traits in IMECG.

Trait	Model	$\sigma_{a}^{2}$	$h_{a}^{2}$	$\sigma_{m}^{2}$	$h_{m}^{2}$	$\sigma_{am}$	$r_{am}$	$\sigma_{c}^{2}$	$h_{c}^{2}$	$\sigma_{e}^{2}$	$\sigma_{p}^{2}$
BW	model1	0.0782 ± 0.0095	0.3884 ± 0.0414							0.1231 ± 0.0071	0.2013
	model2	0.0080 ± 0.0044	0.0345 ± 0.232					0.0427 ± 0.0041	0.0933 ± 0.0351	0.13750.0046	0.2308
	model3	0.0053 ± 0.0032	0.0279 ± 0.0163	0.0438 ± 0.0040	0.0976 ± 0.0201					0.1393 ± 0.0041	0.1883
	model4	0.0247 ± 0.0082	0.1290 ± 0.0415	0.0839 ± 0.0010	0.1218 ± 0.0198	−0.0442 ± 0.0100	−0.0641			0.1268 ± 0.0059	0.1913
	model5	0.0056 ± 0.0034	0.0272 ± 0.0181	0.0271 ± 0.0075	0.0652 ± 0.0209			0.0181 ± 0.0077	0.0433 ± 0.0297	0.1373 ± 0.0042	0.2061
	model6	0.0254 ± 8.183008 × 10^−3^	0.1330 ± 0.0416	0.0850 ± 9.846178 × 10^−3^	0.1220 ± 0.0192	−0.0460 ± 9.997023 × 10^−3^	−0.0660	5.77 × 10^−6^ ± 8.794646 × 10^−3^	8.28 × 10^−6^ ± 0.0222	0.1264 ± 9.846178 × 10^−3^	0.1908
WW	model1	2.0224 ± 0.3822	0.2951 ± 0.0511							4.8318 ± 0.2996	6.8542
	model2	0.2025 ± 0.1318	0.0277 ± 0.0202					0.8063 ± 0.1398	0.1104 ± 0.0213	5.4866 ± 0.1813	7.3017
	model3	0.1611 ± 0.1145	0.0247 ± 0.0175	0.8331 ± 0.1382	0.1278 ± 0.0305					5.5225 ± 0.1728	6.5167
	model4	0.1775 ± 0.1194	0.0272 ± 0.0182	0.5970 ± 0.2269	0.0915 ± 0.03	0.2234 ± 0.1796	0.0343			5.5245 ± 0.1730	6.5225
	model5	0.1768 ± 0.1212	0.0252 ± 0.0185	0.3439 ± 0.2997	0.0490 ± 0.0196			4.94 × 10^−1^ ± 0.3091	7.04 × 10^−2^ ± 0.0005	5.5046 ± 0.1761	7.0133
	model6	0.2637 ± 0.1563	0.0381 ± 0.0237	0.2131 ± 0.1485	0.0308 ± 0.0201	0.2350 ± 0.2655	0.0340	3.73 × 10^−1^ ± 0.2855	5.40 × 10^−2^ ± 0.0310	5.4563 ± 0.1836	6.9142
ADG	model1	374.4621 ± 70.7725	0.2749 ± 0.0480							987.5901 ± 56.4361	1362.0522
	model2	9.9015 ± 16.3606	0.0076 ± 0.12263					231.0019 ± 28.1657	0.1784 ± 0.0025	1053.7089 ± 32.6786	1294.6123
	model3	7.0710 ± 15.0365	0.0054 ± 0.0116	212.8514 ± 27.2987	0.1640 ± 0.0195					1077.8684 ± 31.9376	1297.7909
	model4	14.7989 ± 17.8982	0.0114 ± 0.0138	274.6565 ± 50.5847	0.2118 ± 0.0301	−61.0918 ± 42.0320	−0.0471			1068.6444 ± 32.7585	1297.0080
	model5	7.4566 ± 15.4108	0.0057 ± 0.0119	123.8752 ± 51.7771	0.0954 ± 0.0175			98.0659 ± 53.8055	0.0756 ± 0.03	1068.4145 ± 32.1868	1297.8121
	model6	9.2362 ± 0.1609	0.0071 ± 0.0127	149.9121 ± 0.5036	0.1157 ± 0.0238	−36.2426 ± 25.9512	−0.0280	106.9093 ± 61.5302	0.0825 ± 0.02	1066.3299 ± 31.9792	1296.1449
12W	model1	2.0863 ± 0.8924	0.2192 ± 0.0968							7.4310 ± 0.8226	9.5173
	model2	1.8972 ± 0.9001	0.1944 ± 0.1050					0.2620 ± 0.0205	0.0276 ± 0.03	7.3365 ± 1.2862	9.7578
	model3	1.8972 ± 0.9198	0.1998 ± 0.0895	0.2620 ± 0.0120	0.0276 ± 0.0211					7.3366 ± 1.0272	9.4958
	model4	1.8953 ± 0.8920	0.1996 ± 0.0992	0.1522 ± 0.0198	0.0160 ± 0.0129	0.1100 ± 0.1921	0.0116			7.3381 ± 1.1953	9.4955
	model5	1.8985 ± 0.8919	0.1999 ± 0.1306	0.2610 ± 0.0149	0.0275 ± 0.0300			6.16 × 10^−5^ ± 0.0298	6.46 × 10^−6^ ± 0.0043	7.3353 ± 0.1051	9.4949
	model6	1.8968 ± 0.9054	0.1998 ± 0.1036	0.1658 ± 0.0137	0.0175 ± 0.01974	0.0953 ± 0.2007	0.0100	6.12 × 10^−5^ ± 0.0271	6.43 × 10^−6^ ± 0.0002	7.3365 ± 0.1926	9.4945

Note: $\sigma_{a}^{2},\sigma_{m}^{2},\sigma_{c}^{2},\sigma_{e}^{2}$ and $\sigma_{p}^{2}$ are additive genetic, maternal additive genetic, direct maternal genetic covariance, maternal permanent environmental variance, residual variance, and phenotypic variance, respectively; $h_{a}^{2},h_{m}^{2},r_{am},h_{c}^{2}$ are direct heritability, maternal heritability, direct maternal genetic correlation, and ratio of maternal permanent environmental effect, respectively.

**Table 3:** Likelihood ratios and AIC and BIC test results of different model comparisons.

Traits	Models	AIC	BIC	(−2logL)
BW	model1	−3072.36	−3059.43	−3076.36
	model2	−3177.03	−3157.67	−3183.03
	model3	−3217.29	−3197.89	−3223.29
	model4	−3239.12	−3213.26	−3247.12
	model5	−3223.72	−3197.86	−3231.72
	model6	−3246.21	−3213.88	−3256.20
WW	model1	12,920.58	12,933.26	12,916.58
	model2	12,892.71	12,911.73	12,886.71
	model3	12,896.38	12,915.4	12,890.38
	model4	12,897.23	12,922.59	12,889.27
	model5	12,898.37	12,923.73	12,890.37
	model6	12,899.23	12,930.93	12,889.23
ADG	model1	34,479.91	34,492.6	34,475.92
	model2	34,414.11	34,433.15	34,408.12
	model3	34,416.25	34,435.29	34,410.26
	model4	34,413.19	34,438.57	34,405.2
	model5	34,419.66	34,445.04	34,411.66
	model6	34,418.89	34,450.61	34,408.88
12W	model1	4308.41	4318.79	4304.41
	model2	4310.18	4325.75	4304.18
	model3	4310.18	4325.75	4304.18
	model4	4312.18	4332.94	4304.18
	model5	4312.17	4332.93	4304.17
	model6	4314.17	4340.12	4304.17

Note: AIC—Akaike information criterion; BIC—Bayesian information criterion; −2log L: −2 × log likelihood.

**Table 4:** Likelihood ratios and *x*^2^ test results of different model comparisons.

Model Comparison	df	BW	WW	ADG	12W
model2/model1	1	106.67 ***	29.88 ***	67.78 ns	0.23 ***
model3/model1	1	146.93 ***	26.2 ***	65.66 ns	0.23 ***
model4/model1	2	170.76 ***	27.36 ***	70.72 ns	0.23 ***
model5/model1	2	155.36 ***	26.22 ***	64.26 ns	0.24 ***
model6/model1	3	179.84 ***	27.37 ***	67.04 ns	0.24 ***
model5/model2	1	48.69 ***	−3.66 ns	−3.54 ns	0.014 ns
model6/model2	2	73.17 ***	−2.52 ns	−0.76 ns	0.014 ns
model4/model3	1	23.83 ***	1.16 ns	5.06 ns	0.002 ns
model5/model3	1	8.43 **	0.014 ns	−1.4 ns	0.014 ns
model6/model3	2	32.91 ***	1.15 ns	1.38 ns	0.014 ns
model6/model4	1	9.08 **	−0.002 ns	−3.68 ns	0.012 ns
model6/model5	1	24.48 ***	1.14 ns	2.78 ns	0 ns

Note: ns—non-significant (*p* > 0.05). The means with different letters in each sub-class within a column differ significantly from one another. ** 0.01 < *p* < 0.05 and *** *p* < 0.01.

**Table 5:** Genetic and phenotypic correlations between growth traits in IMEWCG.

Traits	BW	WW	ADG	12W
BW		0.3906 ± 0.0165	−0.7906 ± 0.0074	0.2014 ± 0.0316
WW	0.5529 ± 0.0843		0.3562 ± 0.0185	0.2497 ± 0.0288
ADG	−0.8700 ± 0.2384	0.3809 ± 0.0735		0.0993 ± 0.0992
12W	−0.6256 ± 0.5475	0.3329 ± 0.3408	0.0755 ± 0.2882	

Note: Above the diagonal is the phenotypic correlation coefficient; Above the diagonal is the genetic correlation coefficient.

## Data Availability

The data presented in this study are available from the corresponding authors upon reasonable request. The data are not publicly available due to privacy or ethical restrictions.

## References

[1] Qi Y., Luo J., Han X., Zhu Y., Chen C., Liu J., Sheng H. (2009). Genetic diversity and relationships of 10 Chinese goat breeds in the Middle and Western China. Small Rumin. Res..

[2] Wang R., Liu Y., Shi Y., Qi Y., Li Y., Wang Z., Zhang Y., Zhao Y., Su R., Li J. (2023). Study of Genetic Parameters For Pre-Weaning Growth Traits in Inner Mongolia White Arbas Cashmere Goats. Front. Vet. Sci..

[3] Liu G., Zhao Q., Lu J., Sun F., Han X., Zhao J., Feng H., Wang K., Liu C. (2019). Insights into the Genetic Diversity of Indigenous Goats and Their Conservation Priorities. Asian-Australas. J. Anim. Sci..

[4] Xie Y., Zhang C., Qin Q., Li X., Guo J., Dai D., Wang Z., Zhao Y., Su R., Wang Z. (2023). Proteomics Analysis of Meat to Identify Goat Intramuscular Fat Deposits Potential Biomarkers. Food Anal. Methods.

[5] Liu L., Zhou J., Chen C.J., Zhang J., Wen W., Tian J., Zhang Z., Gu Y. (2020). GWAS-Based Identification of New Loci for Milk Yield, Fat, and Protein in Holstein Cattle. Animals.

[6] Missanjo E., Imbayarwo-Chikosi V., Halimani T. (2013). Estimation of Genetic and Phenotypic Parameters for Production Traits and Somatic Cell Count for Jersey Dairy Cattle in Zimbabwe. ISRN Vet. Sci..

[7] Gebreyohannes G., Koonawootrittriron S., Elzo M.A., Suwanasopee T. (2013). Variance Components and Genetic Parameters for Milk Production and Lactation Pattern in an Ethiopian Multibreed Dairy Cattle Population. Asian-Australas. J. Anim. Sci..

[8] Fitting of Growth Curves and Estimation of Genetic Relationship between Growth Parameters of Qianhua Mutton Merino—PubMed. https://pubmed.ncbi.nlm.nih.gov/38540449/.

[9] Makgopa L.F., Mathapo M.C., Tyasi T.L. (2023). A systematic Review of Estimation of Growth Curve in Goats. Trop. Anim. Health Prod..

[10] Kushwaha B.P., Mandal A., Arora A.L., Kumar R., Kumar S., Notter D.R. (2009). Direct and Maternal (Co)Variance Components and Heritability Estimates for Body Weights in Chokla Sheep. J. Anim. Breed. Genet..

[11] Kgari R.D., Muller C., Dzama K., Makgahlela M.L. (2022). Estimation of Genetic Parameters for Heifer and Cow Fertility Traits Derived from On-Farm AI Service Records of South African Holstein Cattle. Animals.

[12] Molaei Moghbeli S., Barazandeh A., Vatankhah M., Mohammadabadi M. (2013). Genetics and Non-Genetics Parameters of Body Weight for Post-Weaning Traits in Raini Cashmere Goats. Trop. Anim. Health Prod..

[13] Menezes L.M., Sousa W.H., Cavalcanti-Filho E.P., Gama L.T. (2016). Genetic Parameters for Reproduction and Growth Traits in Boer Goats in Brazil. Small Rumin. Res..

[14] Wiggans G.R., VanRaden P.M. (1991). Method and Effect of Adjustment for Heterogeneous Variance. J. Dairy. Sci..

[15] Bezerra A.S., Dos Santos M.A.S., Lourenço-Júnior J.d.B. (2022). Technologies Used in Production Systems for Santa Inês Sheep: A Systematic Review. Front. Vet. Sci..

[16] Jia Y., Jannink J.-L. (2012). Multiple-Trait Genomic Selection Methods Increase Genetic Value Prediction Accuracy. Genetics.

[17] Sgrò C.M., Hoffmann A.A. (2004). Genetic Correlations, Tradeoffs and Environmental Variation. Heredity.

[18] Tarekegne A., Wegary D., Cairns J.E., Zaman-Allah M., Beyene Y., Negera D., Teklewold A., Tesfaye K., Jumbo M.B., Das B. (2023). Genetic Gains in Early Maturing Maize Hybrids Developed by the International Maize and Wheat Improvement Center in Southern Africa During 2000–2018. Front Plant Sci..

[19] Li Z., Gao C., Che F., Li J., Wang L., Cui K. (2024). Trunk Distortion Weakens the Tree Productivity Revealed by Half-Sib Progeny Determination of Pinus Yunnanensis. BMC Plant Biol..

[20] Shi Y., Qi Y., Liu Y., Rong Y., Ao X., Zhang M., Xia Q., Zhang Y., Wang R. (2024). Study of the Influence of Non-Genetic Factors on the Growth and Development Traits and Cashmere Production Traits of Inner Mongolia White Cashmere Goats (Erlangshan Type). Vet. Sci..

[21] Kannan T.A., Jaganathan M., Ramanujam R., Chinnaondi B., Illa S.K., Kizilkaya K., Peters S.O. (2022). Multi-Trait Bayesian Analysis and Genetic Parameter Estimates in Production Characters of Mecheri Sheep of India. Trop. Anim. Health Prod..

[22] Al-Shorepy S.A., Alhadrami G.A., Abdulwahab K. (2002). Genetic and Phenotypic Parameters for Early Growth Traits in Emirati Goat. Small Rumin. Res..

[23] Borg R.C., Notter D.R., Kott R.W. (2009). Phenotypic and Genetic Associations Between Lamb Growth Traits and Adult Ewe Body Weights in Western Range Sheep. J. Anim. Sci..

[24] Campos G.S., Cardoso F.F., Gomes C.C.G., Domingues R., de Almeida Regitano L.C., de Sena Oliveira M.C., de Oliveira H.N., Carvalheiro R., Albuquerque L.G., Miller S. (2022). Development of Genomic Predictions for Angus Cattle in Brazil Incorporating Genotypes from Related American Sires. J. Anim. Sci..

[25] Balasundaram B., Muralidharan J., Murali N., Cauveri D., Raja A., Okpeku M., Thiruvenkadan A.K. (2023). Development of Selection Strategies For Genetic Improvement in Production Traits of Mecheri Sheep Based on a Bayesian Multi Trait Evaluation. PLoS ONE.

[26] Ladeira G.C., de Paiva J.T., de Oliveira H.R., Carrara E.R., Pilonetto F., Freitas F.A.O., de Mattos E.C., Eler J.P., Ferraz J.B.S., de Genova Gaya L. (2021). Random-Effect Meta-Analysis of Genetic Parameter Estimates for Carcass and Meat Quality Traits in Beef Cattle. Trop. Anim. Health Prod..

[27] Hayward L.K., Sella G. (2022). Polygenic Adaptation After a Sudden Change in Environment. Elife.

[28] Passamonti M.M., Somenzi E., Barbato M., Chillemi G., Colli L., Joost S., Milanesi M., Negrini R., Santini M., Vajana E. (2021). The Quest for Genes Involved in Adaptation to Climate Change in Ruminant Livestock. Animals.

[29] Wass A.V., Butler G., Taylor T.B., Dash P.R., Johnson L.J. (2020). Cancer Cell Lines Show High Heritability For Motility but not Generation Time. R. Soc. Open Sci..

[30] Laghouaouta H., Pena R.N., Ros-Freixedes R., Reixach J., Díaz M., Estany J., Armengol R., Bassols A., Fraile L. (2021). A Methodology to Quantify Resilience in Growing Pigs. Animals.

[31] Gagnier J.J., Morgenstern H., Altman D.G., Berlin J., Chang S., McCulloch P., Sun X., Moher D., Ann Arbor Clinical Heterogeneity Consensus Group (2013). Consensus-Based Recommendations for Investigating Clinical Heterogeneity in Systematic Reviews. BMC Med. Res. Methodol..

[32] Barazandeh A., Moghbeli S.M., Vatankhah M., Mohammadabadi M. (2012). Estimating Non-Genetic and Genetic Parameters of Pre-weaning Growth Traits in Raini Cashmere Goat. Trop. Anim. Health Prod..

[33] Rodríguez-Hernández P., Simões J., Arce C., Díaz-Gaona C., López-Fariñas M.D., Sánchez-Rodríguez M., Rodríguez-Estévez V. (2022). Effect of Non-Genetic Factors on Reproduction of Extensive Versus Intensive Florida Dairy Goats. Vet. Sci..

[34] Santos G.V.D., Sousa J.E.R.D., Sarmento J.L.R., Sousa Júnior S.C.D., Sousa W.H.D., Biagiotti D., Santos N.P.D.S., Rêgo Neto A.D.A. (2013). Modelagem Do Efeito Materno e Estimativa de Parâmetros Genéticos Para Pesos Corporais de Caprinos Anglo Nubiano. Rev. Bras. Saúde Produção Anim..

[35] Boujenane I., Diallo I.T. (2017). Estimates of Genetic Parameters and Genetic Trends for Pre-Weaning Growth Traits in Sardi Sheep. Small Rumin. Res..

[36] Hasan F., Jakaria J., Gunawan A. (2014). Genetic and Phenotypic Parameters of Body Weight in Ettawa Grade Goats. Med. Pet..

[37] Meza-Herrera C.A., Menendez-Buxadera A., Serradilla J.M., Lopez-Villalobos N., Baena-Manzano F. (2019). Estimates of Genetic Parameters and Heterosis for Birth Weight, One-Month Weight and Litter Size at Birth in Five Goat Breeds. Small Rumin. Res..

[38] Sharif N., Ali A., Dawood M., Khan M.I.-U.-R., Do D.N. (2022). Environmental Effects and Genetic Parameters for Growth Traits of Lohi Sheep. Animals.

[39] Beyene G.M., Azale T., Gelaye K.A., Ayele T.A. (2021). The Effect of Antenatal Depression on Birth Weight Among Newborns in South Gondar Zone, Northwest Ethiopia: A Population-Based Prospective Cohort Study. Arch. Public Health.

[40] Diego M.A., Field T., Hernandez-Reif M., Schanberg S., Kuhn C., Gonzalez-Quintero V.H. (2009). Prenatal Depression Restricts Fetal Growth. Early Hum. Dev..

[41] Fekadu Dadi A., Miller E.R., Mwanri L. (2020). Antenatal Depression and Its Association with Adverse Birth Outcomes in Low and Middle-Income Countries: A Systematic Review and Meta-Analysis. PLoS ONE.

[42] Davenport M.H., Hayman M. (2022). Physical Activity During Pregnancy: Essential Steps for Maternal and Fetal Health. Obs. Med..

[43] Hsu T.-Y., Cheng H.-H., Lan K.-C., Hung H.-N., Lai Y.-J., Tsai C.-C., Fan W.-L., Li S.-C. (2023). Author Correction: The abundances of LTF and SOD2 in Amniotic Fluid Are Potential Biomarkers of Gestational Age and Preterm Birth. Sci. Rep..

[44] González-Nieto P.F., Alvarado-Olivarez M., Guzmán-Gerónimo R.I., Rodríguez-Landa J.F., Hernández-Salazar L.T. (2024). Effect of Traditional and Non-Traditionally Processed Blue Corn Tortilla Consumption During the Gestation of Rats in the Dentate Gyrus of Pups. Foods.

[45] Igweze Z.N., Ekhator O.C., Nwaogazie I., Frazzoli C., Orisakwe O.E. (2019). Appropriateness of Essentials Trace Metals in Commonly Consumed Infant Formulae in Nigeria. Open Access Maced. J. Med. Sci..

[46] Seyoum G., Persaud T.V. (1995). Influence of Zinc on Ethanol-Induced Placental Changes in the Rat. Histol. Histopathol..

[47] Torres-Cuevas I., Parra-Llorca A., Sánchez-Illana A., Nuñez-Ramiro A., Kuligowski J., Cháfer-Pericás C., Cernada M., Escobar J., Vento M. (2017). Oxygen and Oxidative Stress in the Perinatal Period. Redox Biol..

[48] Bangar Y.C., Magotra A., Yadav A.S. (2020). Estimates of Covariance Components and Genetic Parameters for Growth, Average Daily Gain and Kleiber Ratio in Harnali Sheep. Trop. Anim. Health Prod..

[49] Ofori S.A., Hagan J.K. (2020). Genetic and Non-Genetic Factors Influencing the Performance of the West African Dwarf (WAD) Goat Kept at the Kintampo Goat Breeding Station of Ghana. Trop. Anim. Health Prod..

[50] Dige M.S., Rout P.K., Singh M.K., Dass G., Kaushik R., Gowane G.R. (2021). Estimation of Co (Variance) Components and Genetic Parameters for Growth and Feed Efficiency Traits in Jamunapari Goat. Small Rumin. Res..

[51] Jafaroghli M., Rashidi A., Mokhtari M.S., Shadparvar A.A. (2010). (Co)Variance Components and Genetic Parameter Estimates for Growth Traits in Moghani Sheep. Small Rumin. Res..

[52] Zhang C.-Y., Zhang Y., Xu D.-Q., Li X., Su J., Yang L.-G. (2009). Genetic and Phenotypic Parameter Estimates for Growth Traits in Boer Goat. Livest. Sci..

[53] Latifi M., Razmkabir M. (2019). Estimation of Genetic Trends for Body Weight Traits in Markhoz Goat at Different Ages. Span. J. Agric. Res..

[54] Gholizadeh M., Ghafouri-Kesbi F. (2015). Estimation of Genetic Parameters for Growth-Related Traits and Evaluating the Results of a 27-Year Selection Program in Baluchi Sheep. Small Rumin. Res..

[55] Hızlı H. (2022). Comparison of Different Models for Estimation of Direct and Maternal Genetic Parameters on Body Weights in Awassi Sheep. Arch. Anim. Breed..

[56] Snyman M. (2012). Genetic Analysis of Body Weight in South African Angora Kids and Young Goats. SA J. An. Sci..

[57] Gowane G.R., Chopra A., Prakash V., Arora A.L. (2011). Estimates of (Co)Variance Components and Genetic Parameters for Growth Traits in Sirohi Goat. Trop. Anim. Health Prod..

[58] Rashidi A., Bishop S.C., Matika O. (2011). Genetic Parameter Estimates for Pre-Weaning Performance and Reproduction Traits in Markhoz Goats. Small Rumin. Res..

[59] Magotra A., Bangar Y.C., Chauhan A., Malik B.S., Malik Z.S. (2021). Influence of Maternal and Additive Genetic Effects on Offspring Growth Traits in Beetal Goat. Reprod. Domest. Anim..

[60] Coker M.O., Laue H.E., Hoen A.G., Hilliard M., Dade E., Li Z., Palys T., Morrison H.G., Baker E., Karagas M.R. (2021). Infant Feeding Alters the Longitudinal Impact of Birth Mode on the Development of the Gut Microbiota in the First Year of Life. Front. Microbiol..

[61] Kuh D., Wills A.K., Shah I., Prentice A., Hardy R., Adams J.E., Ward K., Cooper C., National Survey for Health and Development (NSHD) Scientific and Data Collection Team (2014). Growth from Birth to Adulthood and Bone Phenotype in Early Old Age: A British Birth Cohort Study. J. Bone Min. Res..

